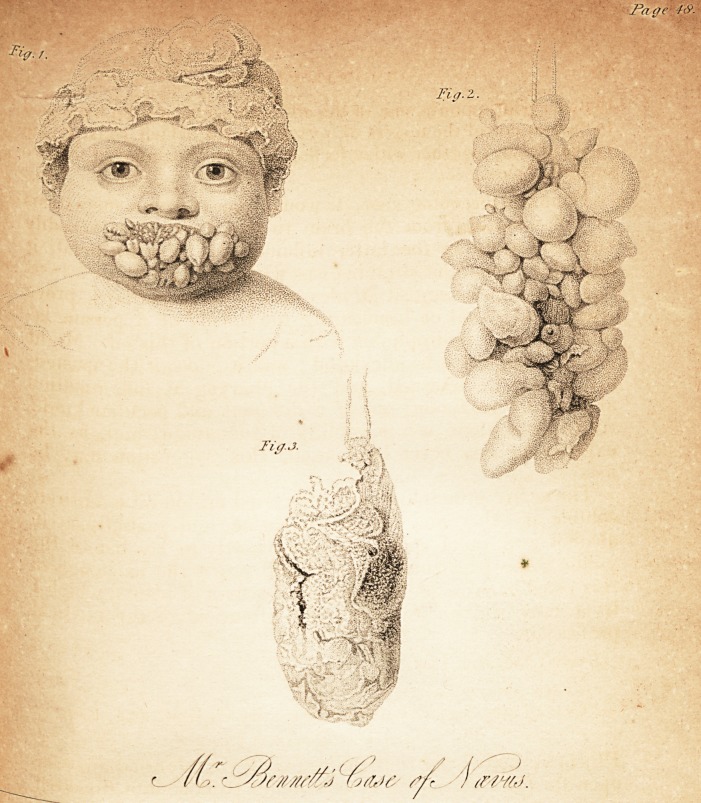# Case of Singular Nævus Maternus

**Published:** 1827-07

**Authors:** G. Bennett


### *Case of singular Nævus Maternus.* By G. Bennett, Esq.

[with an engraving.]

Feeling convinced that the pages of your Journal are ever open for the insertion of facts tending to elucidate disputed points in medical science, I forward the following case of Nævus Maternus, which occurred in the practice of Mr. Baldy, surgeon to the Plymouth Infirmary. The accompanying rough but correct delineations of the extraordinary tumors, as they appeared both before and after excision, will give some idea of their general appearance; whilst in the original many peculiarities are evident, which, if they do not set entirely at rest the question of mental excitement influencing the organisation of the fœtus, must at least be admitted as striking coincidences. I shall only add, that the preparations are in my possession, and I shall be happy to submit them to the inspection of any of your readers who may consider the case as one worthy of further corroboration.

*17, Norfolk-street, Middlesex Hospital;*
*May 2d,* 1827.

Fig. 1.

Fig. 2.

Fig. 3.

Mr. Bennett's Case of Nævus.

Page 50

Mr. Rolfe's Case of Lusus Naturæ.

Engraved for the London Medical & Physical Journal July 1827. — No. 13. New Series.

On the morning of the 19th of March last, Mr. Baldy's attendance was requested on a poor woman, named Hooper, (residing in Lower-street, Plymouth,) then in the pains of labour, and who, shortly after his arrival, was safely delivered of a male child, presenting the following remarkable appearance. The mouth was extended to its utmost limits, and rendered incapable of being closed, by the existence of a cluster of tumors of various sizes, (vide Fig. 1st,) which occupied and projected from the upper and middle part of the tongue, and adhered to the greater portion of the superior surface of that organ. The tumors bore in appearance an astonishing resemblance to a bunch of grapes, (vide Fig. 2d,) not in form alone, but also in colour, being of a yellowish-green hue, and covered with a very delicate pellicle or epidermis; whilst the similitude was rendered still greater by the globular tumors decreasing in size, in the manner so peculiar to a bunch of grapes. Besides this peculiar excrescence, there was yet another upon the same child, and attached to the upper part of the thorax: it consisted of a long portion of integuments, similar, both in size and colour, to the wattle of a turkey-cock, (vide Fig. 3d.)

Six hours after birth, both nævi were removed by Mr. Baldy, at which operation I assisted. Their removal was attended with but little hemorrhage, and no vessels were required to be secured. In the course of a few days after the operation, the little sufferer was able to take the breast, and is now perfectly recovered.

It next became a question of interest, what could have given rise to these peculiar excrescences? Upon my questioning the woman whether she recollected having felt a wish or longing for any particular object during pregnancy, she immediately recalled to mind that she had "wished," or "longed" for grapes, but more particularly on one occasion when she saw a boy eating some; and, still thinking on them when passing the streets, she was continually looking at the grapes in the fruiterer's shops. The only circumstance that can account for the piece of integument resembling the wattle of a turkey-cock is, that, when about four months advanced in pregnancy, going into a neighbour's courtilage, she was frightened by a turkey-cock running after her, as if for the purpose of attack, and, to use her own expression, "turned her blood." I consider it requisite to mention, that the woman, at the time the questions were asked, *was not aware* that any deformity whatsoever existed with respect to the infant; although afterwards, her suspicions being excited, she inquired whether "there were any marks about the child?"

On this subject I have found medical men generally sceptical. I mention this instance as not the only one, but the most extraordinary of the kind that has fallen under my notice.*

* We have examined the preparations, and can bear testimony to the accuracy of the description given of them by Mr. Bennet.—Editor.

## Figures and Tables

**Fig. 1. Fig. 2. Fig. 3. f1:**